# Medical College of Ohio

**Published:** 1850-10

**Authors:** 


*Medical College of Ohio*—H. Willis Baxley, M. D., of Baltimore, Md., has been appointed to the chair of anatomy made vacant by the decease of Prof. Shortwell. Dr. Baxley has been for several years past connected with the Washington University of Baltimore. He sustains a high reputation as an anatomist and lecturer.

